# Evaluation of Analgesic and Antipyretic Activities of *Centratherum anthelminticum* (L) Kuntze Seed

**DOI:** 10.4103/0250-474X.57302

**Published:** 2009

**Authors:** A. Purnima, B. C. Koti, V. P. Tikare, A. H. M. Viswanathaswamy, A. H. M. Thippeswamy, P. Dabadi

**Affiliations:** Department of Pharmacology, KLES's College of Pharmacy, Rajajinagar, Bangalore-560 010, India; 1 Department of Pharmacology, KLES's College of Pharmacy, Vidyanagar, Hubli-580 031, India

**Keywords:** *Centratherum anthelminticum*, analgesic activity, antipyretic activity, brewer's yeast-induced fever model, acetic acid-induced writhing

## Abstract

The study was designed to investigate analgesic and antipyretic activities of petroleum ether and alcohol extracts of *Centratherum anthelminticum* (L) Kuntze (family: Asteraceae) seeds (100 and 200 mg/kg, p.o.) in brewer's yeast-induced fever model in rats, acetic acid-induced writhing and Eddy's hot plate methods in mice. Both petroleum ether and alcohol extracts showed significant decrease in number of writhes in acetic acid-induced writhing and increase in paw licking time to heat stimuli in the hot plate method. The maximum analgesic activity was observed at 90 min after dosing when compared to control. Both the extracts showed significant inhibition of elevated body temperature when compared to corresponding control. These results suggested that the petroleum ether and alcohol extracts possessed analgesic and antipyretic activities.

The plant *Centratherum anthelminticum* (L) is highly reputed in traditional medicine as remedy for leucoderma and other skin diseases. The seeds have a hot sharp taste; acrid, astringent to the bowels, anthelmintic and cure ulcer. The seeds are used as purgative, for asthma, kidney troubles and hiccough, applied in inflammatory swelling, good for sores and itching of the eyes. In Punjab, it is considered as febrifuge i.e. antipyretic. The seeds are also credited with tonic, stomachic and diuretic properties[1].

*Centratherum anthelminticum* (L) Kuntze seeds were reported to contain flavonoids, 2',3,4,4-tetrahydroxychalcone, 5,6,7,4'-tetrahydroxy flavone and 7,3',4'-trihydroxydihy droflavone (butin)[2], sterol-4α-methylvernosterol, vernosterol, avernosterol[3], (24a/R)-stigmasta-7-en-3-one (1), (24a/R)-stigmasta-7,9(11)-dien-3-one (2), (24a/S)-Stigmasta-5, 22-dien-3ß-ol (3) and (24a/S)-stigmasta-7,22-dien-3β-ol[4], brassicasterol, stigmasterol, and an alkaloid, vernonine. Other fractions like stearic, palmitic, myristic, oleic, monohydroxy-oleic acids, linoleic, vernolic acid and resin[5]. Hence the present work has been taken up to substantiate the folklore use of *Centratherum anthelminticum* (L) Kuntze seeds on experimental algesia and pyrexia in mice and rats.

The seeds of *Centratherum anthelminticum* (L) were purchased from local market and authenticated at the Department of Botany, H. S. Kothambri Science Institute, Hubli. The powdered material was subjected to successive extraction in a Soxhlet apparatus using petroleum ether followed by ethanol. The yield was 13.6% w/w and 14.21% w/w for petroleum ether and alcohol extract, respectively.

Wistar rats (150-200 g) and mice (20-30 g) of either sex were selected and kept for one week to acclimatize to laboratory conditions before starting the experiment. They were subjected to standard diet and water *ad libitum*, but 12 h prior to an experiment, the rats were deprived of food but not water. The acute oral toxicity[6] study was carried out as per the guidelines set by Organization for Economic Co-operation and Development (OECD). The study was approved by the Institutional Animal Ethics Committee (IAEC). A dose of 200 mg/kg was taken as effective dose for both petroleum and alcohol extracts of *Centratherum anthelminticum* (L) Kuntze seeds to evaluate analgesic and antipyretic activities.

The mice were divided into six groups of six each. To each group 0.1 ml of 1% acetic acid was injected intraperitonially[7]. Control group rats received saline solution (0.9% w/v, NaCl) 2 ml/kg and standard group of mice received 40 mg/kg ibuprofen. The test groups of mice were treated orally with 100, 200 mg/kg of the petroleum ether and alcohol extract 60 min before acetic acid injection. Number of writhes (abdominal muscle contraction), stretching of the hind limbs and trunk twisting were counted for 10 min after acetic acid injection. Percent inhibition was determined for each experimental group as follows: Percent inhibition = (N-N^t^/N)×100, where N is the average number of writhing of control per group and N^t^ is the average number of writhing of test per group.

The mice were divided into six groups of six each. The test was carried out using Eddy's hot plate apparatus[8], the temperature was set at 55±1^°^. Mice were placed on hot plate and recorded the reaction time in second for licking of hind paw or jumping with cut off time of 15 s. The reaction time following the administration of the test extracts, reference standard pentazocine (5 mg/kg) and control saline vehicle were measured at 0, 30, 90 and 180 min.

Wistar rats were divided into six groups of six rats each. Animals were fevered by injecting 20 mg/kg of 20 % suspension of Brewer's yeast subcutaneously[9]. Initial rectal temperature was recorded. After 18h, animals that showed an increase of 0.3–0.5^°^ in rectal temperature were selected. The test extracts, reference standard paracetamol (150 mg/kg) and control saline vehicle were administered orally and rectal temperature was recorded by digital thermometer 30 min before and 0.5, 1, 2, 3, 4, 5 and 6 h after extracts/ drug administration.

The results were expressed as the mean±SEM. The results obtained from the present study were analyzed using One way ANOVA followed by Dunnett's multiple comparison tests. p<0.05 was used to indicate statistical significance. Data was computed for statistical analysis by using Graph Pad Prism Software.

The mean writhing response of control, standard and test extracts are shown in Table 1. The mean writhing response of standard ibuprofen (40 mg/kg) treated group was found to be 24.67. The test compounds, petroleum ether extract 200 mg/kg, alcoholic extract 200 mg/kg, petroleum ether extract 100 mg/kg and alcoholic extract 100 mg/kg showed mean writhing responses as 27.5, 36.17, 59.83, and 62.83, respectively. Hence number of writhing was less when compared with control 72.33. Ibuprofen inhibited writhing by 65.89%, while with the test compound petroleum ether extract 200 mg/kg, alcohol extract 200 mg/kg, petroleum ether extract 100 mg/kg and alcohol extract 100 mg/kg were found to be 61.98%, 50%, 17.28% and 13.13%, respectively when compared with control. These test compounds were found to be statistically significant. The petroleum ether extract 200 mg/kg and alcohol extract 200 mg/kg were found to be more significant.

**TABLE 1:** EFFECT OF *CENTRATHERUM ANTHELMINTICUM* SEED EXTRACTS ON ACETIC ACID INDUCED WRITHES IN MICE

Treatment	Dosage (mg/kg)	Number of writhing	% of Inhibition
Control	Saline	72.33±3.35	
Ibuprofen	40	24.67±1.25*	65.89
Pet ether extract	100	59.83±2.21*	17.26
Pet ether extract	200	27.50±1.38*	61.98
Alcohol extract	100	62.83±2.25*	13.13
Alcohol extract	200	36.17±1.47*	50.00

Values expressed as mean±SEM, n=6 animals in each group. The results were analyzed using One way ANOVA followed by Dunnett's multiple comparison tests.

* p<0.05 was used to indicate statistical significance when compared to control.

In the hot plate method, pentazocine showed an increase in reaction time to the heat stimulus, which was found to be significant at 90 min after treatment when compared with control (Table 2). The petroleum ether and alcohol extracts at a dose of 200 mg/kg showed significant increase in reaction time i.e. 8.12 s and 12.82 s, respectively at 90 min. Petroleum ether extract and alcohol extract at a dose of 100 mg/kg showed 4.52 seconds and 6.87 s increase in reaction time at 90 min when compared to control (2.97 s). These increases were found to be statistically significant. Hence alcohol extract 200 mg/kg was found to be more effective compared to petroleum ether extract.

**TABLE 2:** EFFECT OF *CENTRATHERUM ANTHELMINTICUM* SEED EXTRACTS ON EDDY'S HOT PLATE TEST IN MICE

Treatment	Dosage (mg/kg)	Paw licking or jumping in seconds			
		
		0 min	30 min	90 min	180 min
Control	Normal saline	2.703±0.22	2.863±0.28	2.937±0.21	2.868±0.20
Pentazocine	5	2.817±0.18	10.68±0.62*	13.50±0.37*	10.95±0.34*
Pet ether extract	100	2.728±0.20	3.763±0.15	4.522±0.21*	3.988±0.41
Pet ether extract	200	2.792±0.13	5.022±0.35*	8.108±0.23*	7.480±0.33*
Alcohol extract	100	2.707±0.21	4.458±0.24*	6.870±0.29*	5.977±0.28*
Alcohol extract	200	2.713±0.14	8.865±0.37*	12.81±0.39*	11.12±0.44*

Values expressed as mean±SEM, n=6 animals in each group. The results were analyzed using One way ANOVA followed by Dunnett's multiple comparison tests.

* p<0.05 was used to indicate statistical significance when compared to control.

The results of the antipyretic effect of the test compounds, standard (paracetamol 150 mg/kg) and control are presented in Table 3. The paracetamol as well as petroleum ether and alcohol extracts at dose of 200 mg/kg started showing effective antipyretic activity after 1h of postdosing, while petroleum ether extract 100 mg/kg and alcohol extract 100 mg/kg reduced temperature after 2 h, when compared with control. Antipyretic activity was observed up to 4 h after paracetamol and test extracts administration.

**TABLE 3:** EFFECT OF *CENTRATHERUM ANTHELMINTICUM* SEED EXTRCTS ON BREWER'S YEAST INDUCED PYREXIA IN RATS

Treatment	Dosage mg/kg	Rectal temperature (°)								
		
		Before yeast	18 h after	Temperature after treatment						
		
				30 min	1 h	2 h	3 h	4 h	5 h	6 h
Control	Normal saline	37.2±.1	37.9±.1	38.1±.1	38.1±.1	38.3±.1	38.4±.1	38.5±.04	38.6±.05	38.7±.04
Paracetamol	150	37.5±.1	38.3±.2	38.1±.2	37.7±.2*	37.5±.1*	37.3±.1*	37.5±.1*	37.8±.1	38.1±.1
Pet ether extract	100	37.4±.1	37.9±.1	38.0±.1	37.8±.1	37.6±.1*	37.5±.1*	37.6±.1*	37.8±.04	38.1±.1
Pet ether extract	200	37.3±.1	38.1±.1	38.0±.2	37.6±.1*	37.5±.1*	37.4±.1*	37.5±.1*	37.9±.05	38.1±.04
Alcohol extract	100	37.6±.1	38.2±.1	38.3±.1	38.2±.1	37.9±.1*	37.8±.1*	37.9±.1	38.3±.1	38.4±.04
Alcohol extract	200	37.3±.1	37.9±.1	37.7±.1	37.6±.1*	37.5±.1*	37.4±.1*	37.5±.03*	37.7±.04	38.0±.1

Values expressed as mean±SEM, n=6 animals in each group. The results were analyzed using One way ANOVA followed by Dunnett's multiple comparison tests.

* p<0.05 was used to indicate statistical significance when compared to control.

The present investigations suggested that petroleum ether and alcohol extracts showed significant analgesic effect in chemical and mechanical induced pain models. Acetic acid-induced writhing and Eddy's hot plate induced thermal stimulation are models of pain that mainly involve peripheral and central mechanisms, respectively. Antinociceptive effect observed in both these models with *Centratherum anthelminticum* (100 and 200 mg/kg) extracts indicates the involvement of both peripheral and central mechanisms. The acetic acid-induced writhing has been associated with increased level of PGE_2_ and PGF_2α_ in peritoneal fluids as well as lipoxygenase products[10]. The present results revealed that a significant reduction in acetic acid-induced writhing strongly suggests that the mechanism of both the petroleum ether and alcohol extracts may be linked partly to cyclooxygenase[11] and/or lipooxygenase.

In the antipyretic test, both extracts markedly decreased the rectal temperature of pyretic rats. This postulation is supported by the antipyretic effect of the extract, evidenced by its impact on the pathogenic fever induced by the administration of a yeast injection. Its etiology includes production of prostaglandins in central nervous system, which is the final common pathway responsible for fever induction[12]. Inhibition of prostaglandin synthesis could then be the possible mechanism of antipyretic actions of these extracts as that of paracetamol[13]. Thus the present study concludes that the petroleum ether and alcohol extracts have analgesic and antipyretic activities in animals at the doses of 100 mg/kg and 200 mg/kg.
